# Simulation analysis of the evolution law of creep rupture crack extension in X-fractured rock body

**DOI:** 10.1038/s41598-024-65818-3

**Published:** 2024-06-27

**Authors:** Na Zhao, Shuai Wei, Laigui Wang, Jingyi Sun

**Affiliations:** School of Mechanics and Engineering, Liaoning Technical University, Fuxin, 123000 China

**Keywords:** X-fractured, Crack extension, GDEM, Creep damage, Natural hazards, Solid Earth sciences, Petrology

## Abstract

Creep is the macroscopic manifestation of the process of generation, expansion, and penetration of microscopic cracks in a rock body. In this study, the GDEM continuous–discontinuous numerical simulation software was used to model a rock body containing X-fractured for the purposes of exploring creep crack expansion and rupture in the rock body, analyzing the effects of various factors on X-fractured the rock body under the rule of change of the creep curve, and assessing the influences of the intersection angle of the fracture and other factors on the non-parallel fractured rock body on the creep rupture process. The results show that an X-fractured rock body exhibits a mixed tensile–shear damage mode, with tensile damage being the main type of damage. In the isotropic creep stage of a rock body with X-fractured , the steady-state creep rate initially increases and then decreases as the sub- fracture length increases, with the change of the fracture angle of the creep rate of the w-type; the sub-fracture length of h is 14 mm, the rock body is the first to enter into the accelerated creep stage, for the different fracture intersection angle of the rock body For the rock mass with different fracture angles, the time sequence of entering the accelerated creep stage is consistent with the creep rate; when the fracture intersection angle is 45°, and the sub-fracture length is 12 mm, the rock mass has the largest degree of fragmentation, which has a significant impact on the creep damage; after using a single variable processing, it is found that the fracture intersection angle, the sub-fracture length and other factors compared to the fracture intersection angle has a greater impact on the creep damage of the X-fractured rock body. This paper can provide theoretical basis and reference for the study of rock engineering creep damage law and mechanical properties of X-fractured rock body.

## Introduction

A large number of cracks exist within natural rock bodies, and the presence of cracks has a large impact on the mechanical properties of rocks^1^. For cracked structures, the internal rock mainly exists in the form of cross-fractured formation^2^, and the macroscopic fracture and destabilization of this type of rock body under long-term creep loading is closely related to the distribution of internal cracks and the generation, expansion and penetration of cracks. The crack extension form, deformation, response and law of rock structure under different engineering environments are not the same, which will be directly related to the stability of rock structure engineering, and it is very easy to induce deformation damages such as sliding of mine slope body, resulting in engineering accidents.

Rock deformation is related to factors such as stress and time. A continuous increase in deformation under a constant load is called creep. The main cause of rock creep rupture is internal microcrack initiation, expansion, and penetration until macroscopic damage occurs. Cracks are randomly distributed within natural rock bodies. When the rock body is subjected to creep load, the existence of cracks reduces the bearing capacity of the rock body, resulting in deformation and destabilizing damage. The random distribution of fracture within the rock makes them difficult to analyze theoretically or study experimentally. Parallel fracture have been simulated^3^, Numerical simulation was used in this study to examine the creep rupture process of rock bodies with non-parallel fractured. The study results have far-reaching significance for the prevention and management of engineering geologic hazards.

The creep rupture process in fractured rock bodies has been studied extensively. Chao et al.^4–6^ carried out creep tests on single-fracture sandstone at different angles and considered the creep characteristics of hard rock under different characteristic stress intervals to explore the creep characteristics of intact rock and fracture-containing rock. Wang et al.^7^ conducted creep experiments on intact granite and granite containing different prefabricated cleavage angles under different stress conditions and established a consistent nonlinear creep model based on the experimental results, which takes into account the initial damage caused by fracture. Che et al.^8^ conducted triaxial creep tests on sandstone with different cleavage inclinations under freezing environment, and established by fractional-order calculus, the cleavage sandstone nonlinear viscoelastic-plastic constitutive equations. Shiet et al.^9^. carried out graded-loading creep testing on red sandstone containing a single fissure and analyzed the specimen’s behavior under creep loading from a macro-fine viewpoint using acoustic emission, digital scattering, and SEM techniques. Li et al.^10^ introduced damage variables into constitutive relations and creep equations based on Kachanov’s creep damage theory to describe the creep process in a fractured rock body and derived an equation for the evolution of the damage variables over time during the creep process. Zhao^11^ established an equivalent Burgers model for creep fracture in a fractured rock body by introducing equivalent stresses and proposed a double Burgers model, which was used in the creep test. Li et al.^12^. carried out creep mechanics testing on saturated frozen Cretaceous sandstone under different peripheral pressure conditions, drew on classical modeling ideas based on a Riemann–Liouville-type integral function to establish a five-element nonlinear creep damage constitutive equation considering low temperature-damage-stress coupling, and carried out validation of the model.

Numerical simulation has the advantages of being lower in cost than experimental testing, permitting intuitive analysis of the results, and facilitating the comparison of multiple schemes. Wang et al.^13^ proposed a transient creep model based on mesoscale damage intrinsic structure to numerically simulate the transient creep of inhomogeneous sandstone, and compared with the test results to validate the creep model. Zhao et al.^11^ established an equivalent Burgers model for creep fracture offracture rock by introducing isostress, and proposed a dual Burgers model to study the creep behavior of fractured rock, and the proposed dual Burgers model was embedded into FLAC3D using the Fish function. In addition, the proposed dual Burgers model was embedded in FLAC3D using the Fish function. Liu et al.^14^ established a numerical simulation of a biaxial Hopkinson test system by using the finite element-discrete element model coupling method, and investigated the dynamic mechanical properties and damage characteristics of cross-fractured rocks with different nodal distributions under peri-compression. Wang et al.^15^ used PFC5.0 software to perform a seepage-fracture coupling simulation, analyzed the crack extension law of combined coal rock under the action of seepage-flow coupling load induced by the mining process, and investigated the pressure gradient distribution in the fluid grid. Zhang et al.^16^ investigated the effect of temperature on the microdamage and macroscopic force characteristics of brittle inhomogeneous rocks based on a coupled finite element-discrete element numerical modeling method (FDEM) and proposed a preheated uniaxial loading model.

Previous studies on fracture in fractured rock bodies under creep loading have focused mainly on crack propagation from experimental, theoretical, and numerical simulation perspectives. Few studies have analyzed the creep rupture of rock bodies with non-parallel fracture. In this study, the continuous–discontinuous GDEM software was used to simulate the creep rupture process of an X-fractured rock body; analyze the process of crack initiation and expansion to the point of occurrence of macroscopic creep damage in the rock body, and provide a theoretical basis for creep damage and creep management in rock engineering.

## Principles of modeling in continuous–discontinuous numerical simulation software GDEM

### Modeling principles

**Figure 1:**
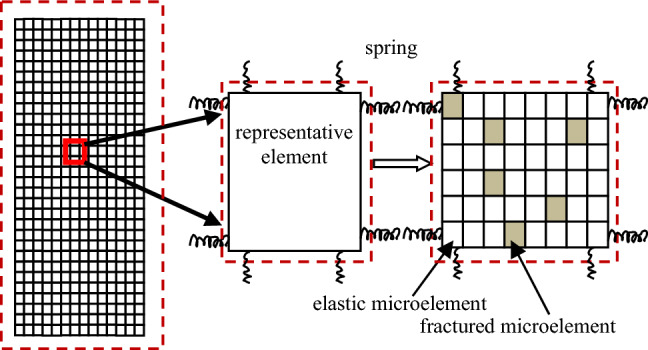
Continuous–discontinuous calculation principle.

The continuous–discontinuous numerical simulation software (GDEM) is a discrete element computational model based on the mechanics of continuous media. (Full name: Gdem(c)Numerical Simulation Integrated Development Platform-Version Block-Dyna 1.0 version number : 1.0.60.1730 Block-Dyna 1.0 URL:DAS.2.10.0.2.msi). The model combines finite elements and discrete elements and performs finite element calculations within the block and discrete element calculations on the block boundaries to simulate the deformation damage and motion characteristics of materials in continuous and discontinuous states. The intrinsic cause of the deformation and rupture of a rock mass is its internal inhomogeneity, as shown in Fig. 1. The Continuous–discontinuous model of the rock mass consists of characterization elements containing elastic and rupture microelements connected by springs. The characterization elements differ in their elastic moduli depending on the internal composition of their microelements. Because of the different elastic moduli of the characterization elements, the springs break during the computational process, and the breakage of the connections between the characterization units simulates the initiation, extension, and penetration of microscopic cracks, thus representing the evolution of macroscopic fracture during the creep and fracture of the rock.

The equations of the continuous–discontinuous computational model are as follows:

1 $$ \begin{aligned} & \int {\left[ {\rho \frac{{{\text{d}}v}}{{{\text{d}}t}} + \frac{\partial }{{\partial x_{j} }}\left( {\lambda \frac{{\partial u_{i} }}{{\partial x_{j} }}\delta_{ij} + G\frac{{\partial u_{i} }}{{\partial x_{j} }}} \right) - C_{m} v - C_{k} \frac{{\partial v_{i} }}{{\partial x_{j} }} + f_{i} } \right]} {\text{d}}v \\ & \quad + \int {\left[ {\overline{T} - \left( {\lambda \frac{{\partial u_{i} }}{{\partial x_{j} }}\delta_{ij} + G\frac{{\partial u_{i} }}{{\partial x_{j} }}} \right)n_{j} } \right]} {\text{d}}S = 0 \\ \end{aligned} $$

The kinetic-equilibrium differential equation of the cell is obtained from the following kinetic equilibrium equation:

2 $$ \sigma_{ij,j} + f_{i} - \rho \ddot{u}_{i} - \mu \dot{u}_{i} = 0 $$

The internal force applied to a unit node should be equal to the unit deformation can be partialized with respect to the displacement of the node, i.e.,

3 $$ F_{i}^{e} = \frac{{\partial \Pi_{e} }}{{\partial u_{i} }} = K_{ij}^{e} u_{j} $$

The Lagrange equation can be written as follows:

4 $$ \int_{V} {\rho \ddot{u}_{i} } {\text{d}}V + \int_{V} {\mu \dot{u}_{i} } {\text{d}}V + F_{i}^{e} = \int_{V} {f_{i} } {\text{d}}V + \int_{S} {\overline{T}_{i} {\text{d}}S} $$

The kinetic equation of the final unit is as follows:

5 $$ M\ddot{u}(t) + C\dot{u}(t) + Ku(t) = F(t) $$

In Eq. 5, $\ddot{u}(t)$_,_ $\dot{u}(t)$_,_ and *u(t)* are the acceleration column, velocity column, and displacement column, respectively, for all nodes in the cell, and *M*, *C*, *K*, and *F(t)* are the cell mass matrix, damping matrix, stiffness matrix, and node external load column, respectively.

**Figure 2:**
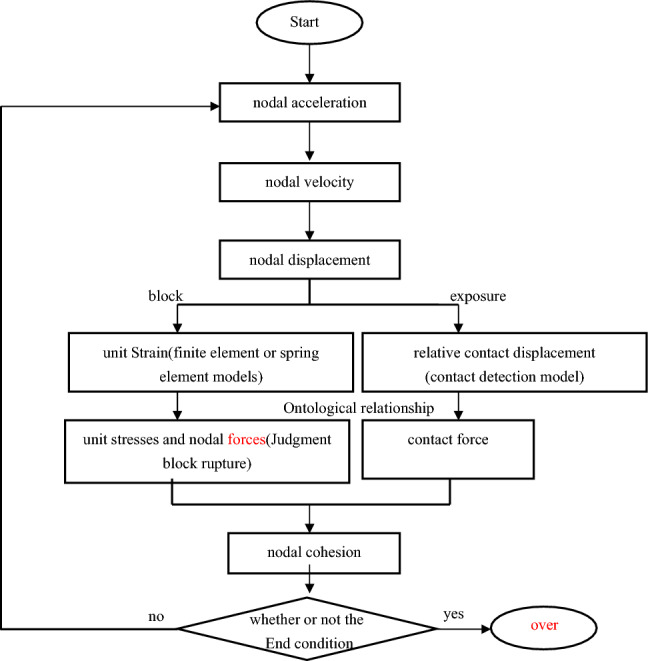
Flow chart.

Unlike the finite element method, in which the overall stiffness matrix is solved, the continuous–discontinuous cell method involves dynamic relaxation without the need to form the overall stiffness matrix after establishing the dynamic solution equations of the cells. The dynamic relaxation method makes the initially unbalanced vibration system gradually decay to the equilibrium position by introducing a damping term in the dynamic calculation, which is a display method to transform the static problem into a dynamic problem for solution. The basic flow of the method is shown in Fig. 2 and summarized below.

(1) Starting from a known initial state, at the end of each time step (e.g., the nth step), all cells in the computational region are fixed.

(2) Calculate the spring force $F_{n}^{S}$ at each cell node. The sum of $F_{n}^{S}$ and the external forces $F_{n}^{e}$ is the combined nodal external force.

6 $$ F_{n} = F_{n}^{S} + F_{n}^{e} $$

(3)According to Eq. 6, the unbalanced force $\left\{ {F_{n}^{\Gamma } } \right\}$ at each unit node is calculated.

7 $$ F_{n}^{\Gamma } = F_{n} - C\dot{u}_{n} - Ku_{n} $$

(4) Based on the unbalanced forces at the nodes on each block, the acceleration of each of the nodes is calculated.

8 $$ a_{n} = M^{ - 1} F_{n}^{\Gamma } $$

(5) According to the acceleration and time step, relax all the nodes at the same time.

9 $$ \dot{u}_{n + 1} = \dot{u}_{n} + a_{n} \Delta t,\quad u_{n + 1} = u_{n} + \dot{u}_{n + 1} \Delta t $$

(6) Fix all nodes at the new position, and loop the next iteration until the exit condition is satisfied.

### Numerical example

**Figure 3:**
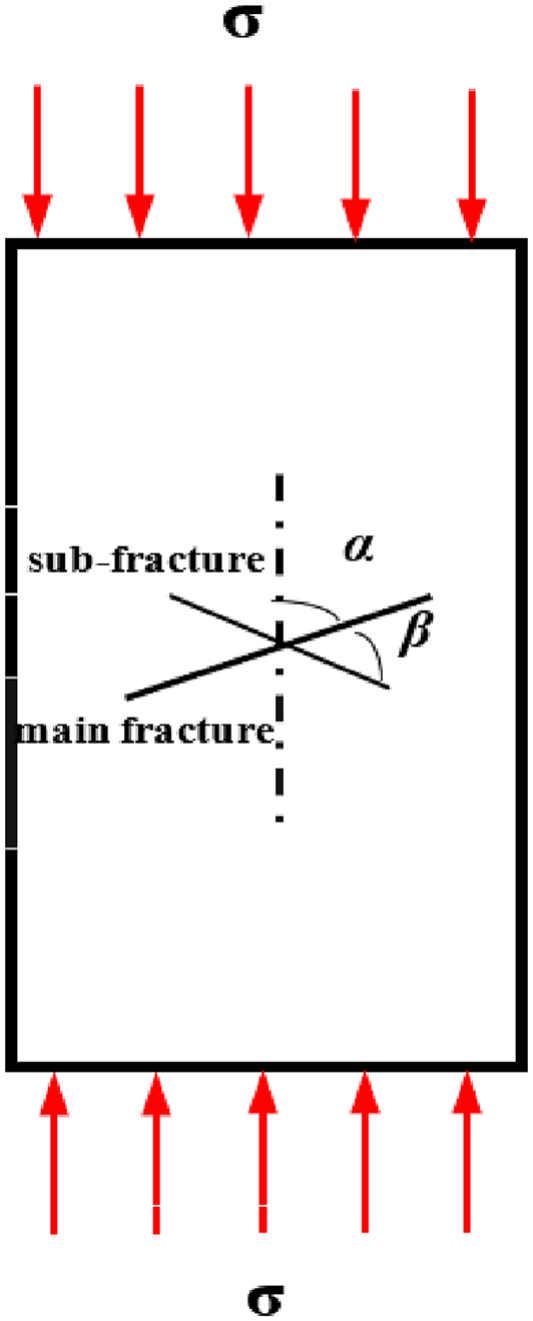
Comparison model diagram.

**Figure 4:**
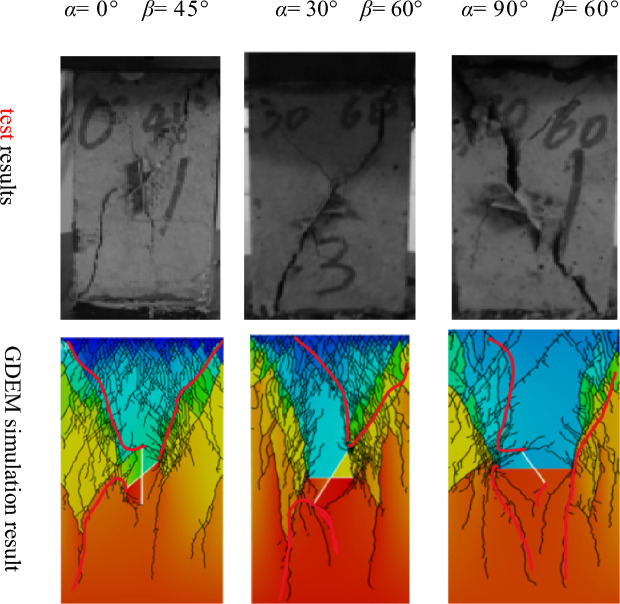
test and X-fractured rock simulation results under GDEM.

In recent years, many researchers have used the discrete element method to carry out simulation studies of the damage mechanism and rupture evolution process of rock bodies and have compared the results with experimental results to ensure the feasibility of simulating the rock body rupture process by the discrete element method^17–19^. As Fig. 3 shows, the rock body considered in this study was one with an X-fractured. The length of the main fracture was 30 mm, the length of the sub-fracture was 20 mm, and the width of the fracture was 0.6 mm. Various rock rupture cloud diagrams can be obtained for various fracture angles and a given set of material parameters and loading stress values. The results can be compared with experimental results obtained by Zhang et al.^20^, as shown in Fig. 4.

The results of the t test and GDEM numerical simulation show that when *α* = 0° and* β* = 45°, the upper end of the main fracture produces wing creaks and expands to the top of the specimen in the direction of the maximum principal stress, the upper and lower ends of the sub-fracture produce wing cracks, and a large number of secondary cracks appear over time. When *α* = 30° and* β* = 60°, the upper end of the main fracture produces two wing cracks and expands to the end of the specimen, and the lower end produces shear cracks. When *α* = 30° and *β* = 60°, two wing cracks are generated at the upper end of the main fracture and extend to the end of the specimen, shear cracks are generated at the lower end of the specimen, and the whole specimen exhibits tensile damage. When *α* = 90° and *β* = 60°, the shear cracks extend in the axial loading direction of the main fracture and the upper end of the sub-fracture, cracks are generated in the process of extension, and the whole specimen exhibits shear damage.

The crack expansion pattern simulated using GDEM is similar to that observed in the test results, and the crack initiation and expansion process can be clearly observed. On this basis, the continuous–discontinuous numerical simulation software GDEM was judged to be suitable for use in simulating the rock crack extension evolution process.

## Simulation of creep rupture of X-fractured rock mass

### Computational model

**Figure 5:**
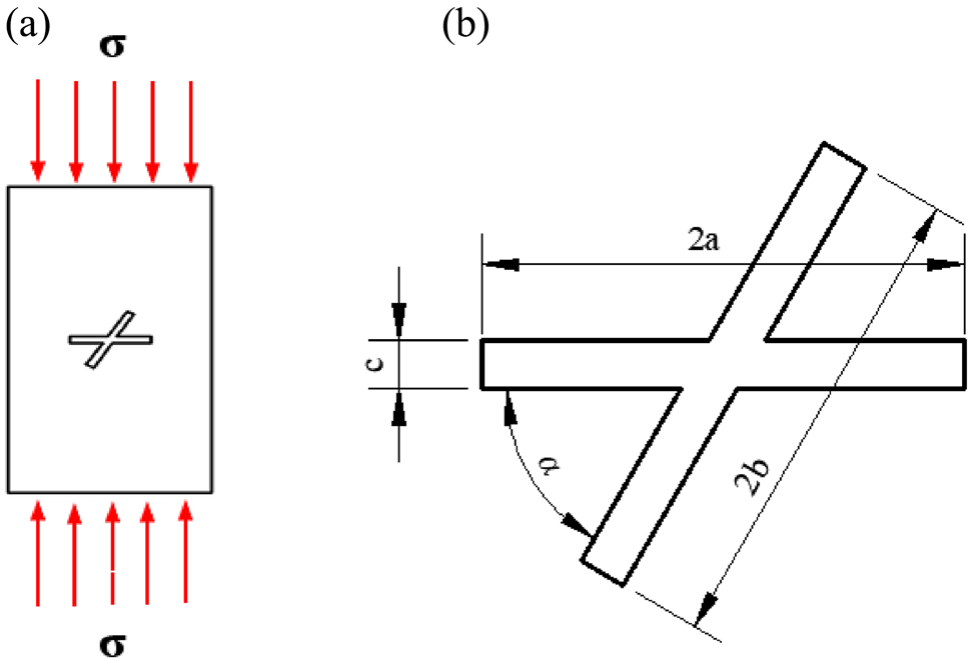
Numerical calculation model of X-fracture. (**a**) X-fracture axial force diagram. (**b**) Localized enlarged view of X-fracture.

The crack extension of the rock body under constant load conditions was numerically simulated using the GDEM software. The microstructure of the rock has an obvious effect on its fracture mechanism^21^, and the computational model is shown in Fig. 5 for a non-homogeneous sandstone containing X-fractured rock mass. The main fracture length 2a is 20 mm, the main fracture inclination angle is 0°, the main fracture and sub-fracture widths c are both 2 mm, 2b is the sub- fracture length, and* α* is the intersection angle between the main fracture and sub-fracture. The size of the model is 50 mm × 100 mm, and different fracture intersection angles and sub-fracture lengths are selected for calculation. The experimental program is shown in Table 1.

**Table 1:** Calculation scheme for X-fractured rock bodies.

Scheme	Intersection angle of fractures*α*/ (°)	Sub-fracture length *2b* (mm)
Scheme 1	30	20
	45	20
	60	20
	75	20
	90	20
Scheme 2	60	12
	60	14
	60	16

Compared with traditional numerical simulation software, the ontological model of the Continuous–discontinuous method is more consistent with the whole process of rock fracture. The block unit is discretized into a spring system, and the spring coefficient is obtained through the energy-generalized function of the spring system to solve the deformation and stress of the unit using the spring stiffness directly. The results of the continuum calculations are consistent with those of finite elements. On this basis, the composite Mohr–Coulomb criterion and maximum tensile stress are introduced to determine the unit rupture state and rupture direction. The internal and boundary rupture of the unit are realized by local block cutting, simulating crack formation and expansion. The basic mechanical parameters of the sandstone material modeled were determined by reviewing the relevant literature and are shown in Table 2.

**Table 2:** Parameters of basic types of numerical simulations.

Material density (kg m^3^)	Tensile strength (MPa)	Internal friction angle (°)	Elastic modulus (MPa)	Cohesion (MPa)	Dilation angle (°)	Poisson’s ratio
2500	3	40.0	1e4	10	10.0	0.25

For discrete elements of a block of deformable bodies, the tangential and normal stiffnesses can be viewed as contact surface stiffnesses. To maintain the continuity of contact in the continuous–discontinuous model, for the spring that connects the characterizing elements in the model, the characteristic stiffness of the contact surface is generally set to 10 or 100 times the characteristic stiffness of the unit, and the numerical calculation is terminated when there is a penetrating fracture in the rock mass or when the calculation cannot converge. The mechanical parameters of the contact surface are shown in Table 3.

**Table 3:** Mechanical parameters of rock contact surfaces.

Normal stiffness (Pa m)	Tangential stiffness (Pa m)	Cohesion (MPa)	Tensile strength (MPa)	Friction angle (°)
5e13	5e13	10	0.4	30.0

### Creep rupture crack extension evolution of X-fractured rock bodies

**Figure 6:**
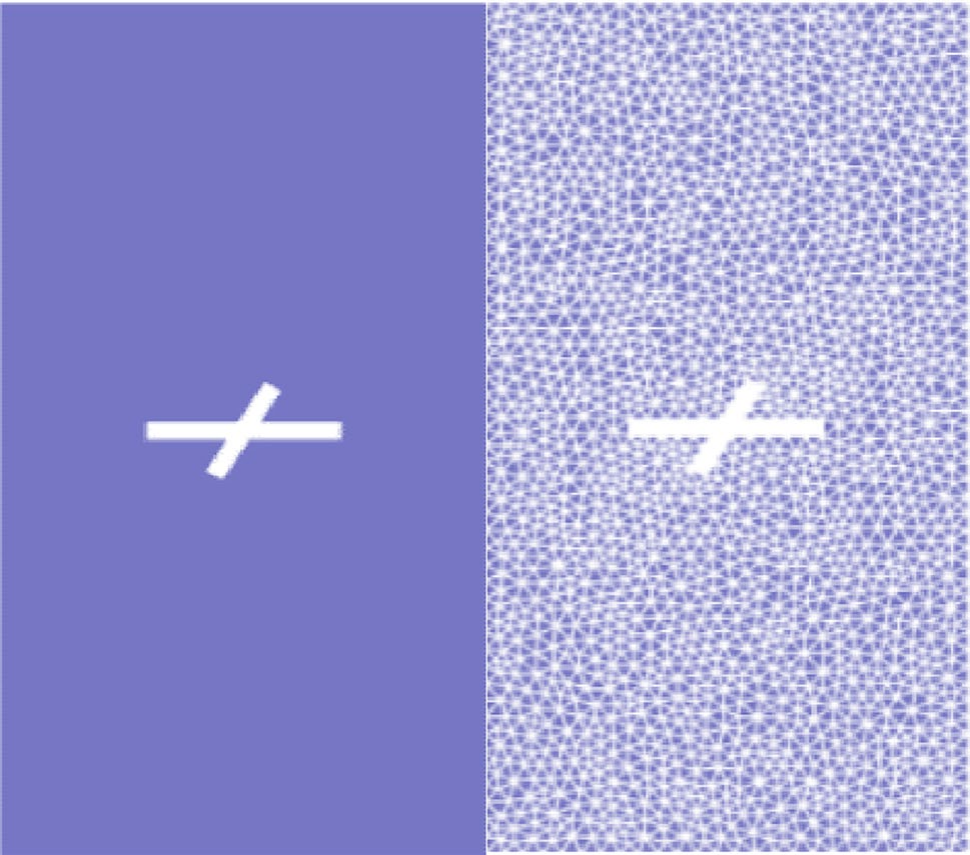
X-fractured model and meshing.

Figure 6 shows the simplified model of the X-fractured rock body established using the GDEM software, with a fracture intersection angle of 60° and a sub-fracture length of 12 mm. The model was meshed using triangles, and mesh refinement was performed at the tip of the fracture to simulate crack initiation better. The model consists of 1,816 nodes and 3,534 meshes. The lower boundary of the model is fixed, and a 3 MPa surface load is applied at the upper end. The computation time starts from zero.

**Figure 7:**
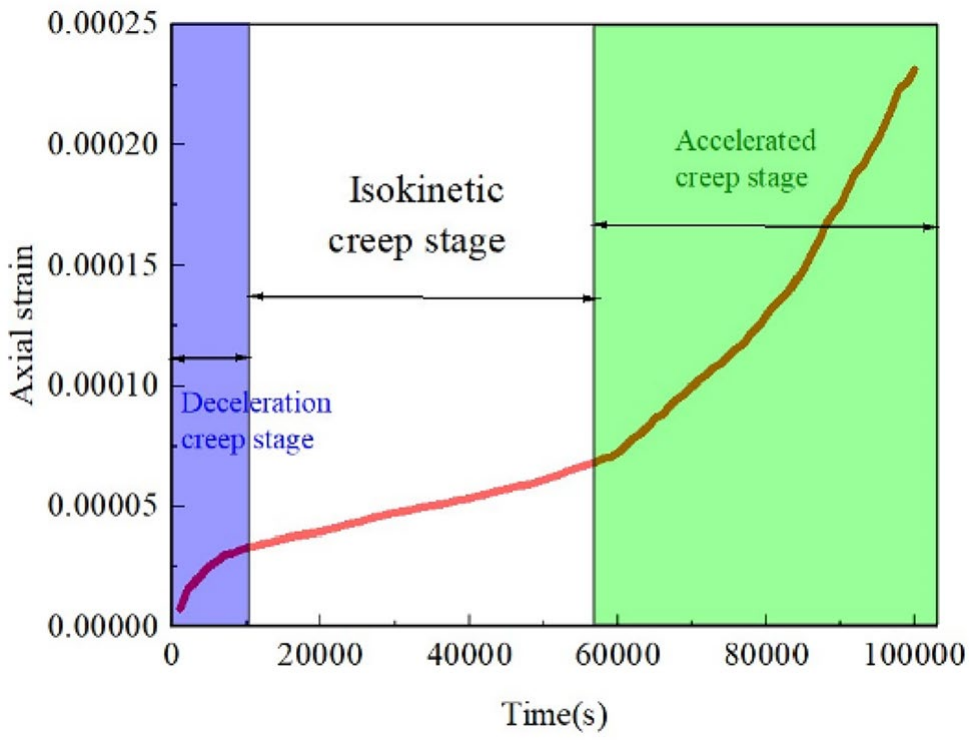
Creep deformation curves of X-fractured rock bodies.

**Figure 8:**
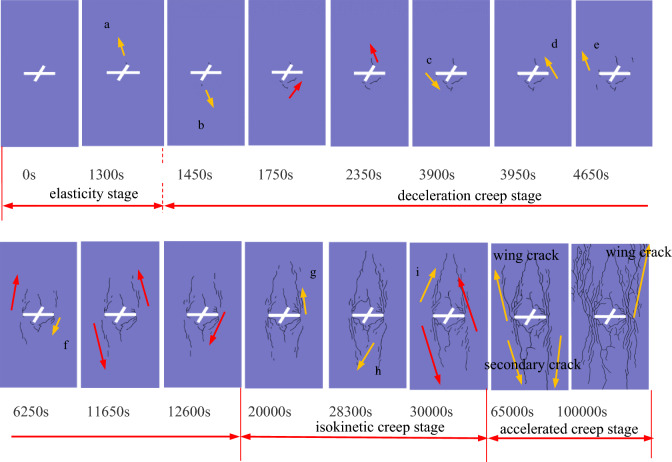
Creep rupture crack extension process in X-fractured rock.

Figures 7 and 8 show the creep rupture process of the X-fractured rock body. The rock mass creep is roughly divided into three stages: decelerated creep stage, isotropic creep stage, and accelerated creep stage. Due to the existence of natural pores inside the fracture, before entering the deceleration creep stage, there will be a short elastic deformation, with the increase of time, cracks will be generated at the end of the initial fracture; isokinetic creep stage, cracks are further extended, extension, macroscopic deformation increases; accelerated creep stage, the cracks penetrate through the formation of macroscopic fracture surfaces, at this time, the rock still has a certain bearing capacity, and thereafter, macroscopic deformation is accelerated, the creep The rate of creep gradually increases.

Wing cracks and secondary cracks are produced at the tips of prefabricated fractures when the rock is loaded. Wing cracks, also called primary cracks or first order cracks, arise as a result of tensile action at the tip of the fracture, followed by a steady expansion in the direction of the maximum principal stress. Secondary cracks are shear cracks that also sprout from the tip of the prefabricated crack and begin with a steady expansion^22^.

0 s to 1300 s, the process is in the elastic stage, no cracks are generated; 1300 s, cracks a are generated at the upper end of the sub-fracture; 1450 s, cracks b are generated near the lower end of the sub-fracture; 1750s, cracks a are expanding along the direction of stress loading, and the number of cracks at the lower end of the sub-fracture is increasing; between 3900 and 4650 s, wing cracks d and e are generated at the ends of the main fracture, and secondary cracks c are generated; when loading time reaches 12600 s, the wing cracks at the ends of the main fracture expand continuously, and secondary crack f occurs at the right end of the crack. time reaches 12600 s, the wing cracks at both ends of the main fracture continue to expand, and s sub-fracture appear at the right end of the crack f. Between 20000 and 30000 s of loading, new cracks g and i appear near the wing cracks at the end of the main fracture, and the cracks near the sub-fracture are connected with the secondary cracks c and f produced by the main crack; at 65000 s, the wing cracks and secondary cracks produced by the main fracture continue to extend along the end of the specimen; at 100,000 s, a clear macroscopic damage zone is finally formed.

In the deceleration creep stage, cracks continue to sprout and expand but do not penetrate. In the isotropic creep stage, cracks sprout and expand, stress is released, and the deformation rate decreases.

## Factors influencing X-fracture creep rupture

### Effect of fracture intersection angle on creep rupture of rocks

**Figure 9:**
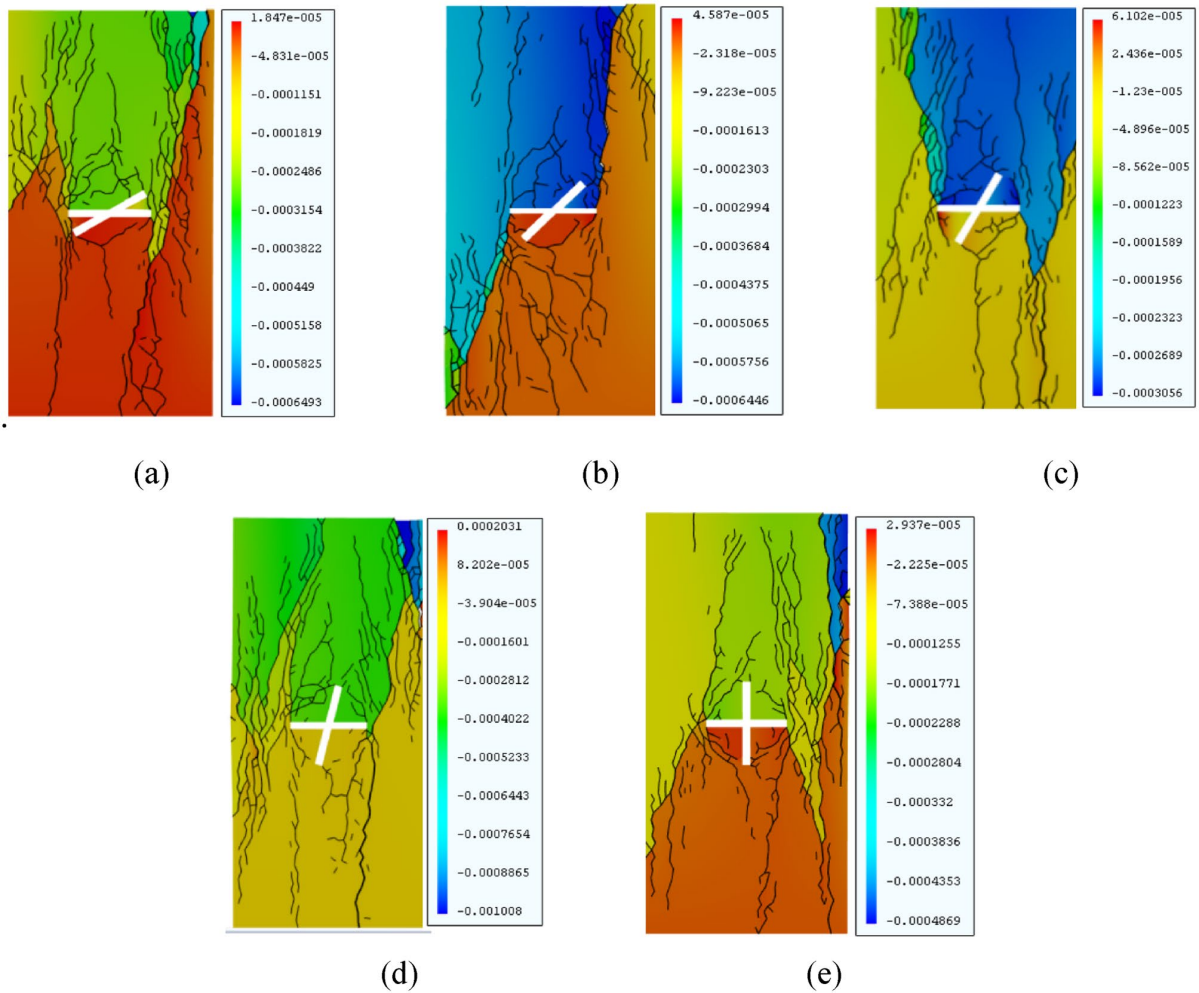
Equivalent displacement maps of rock bodies with different fracture intersection angles under GDEM. (**a**) α = 30°. (**b**) α = 45°. (**c**) α = 60°. (**d**) α = 75°. (**e**) α = 90°.

**Figure 10:**
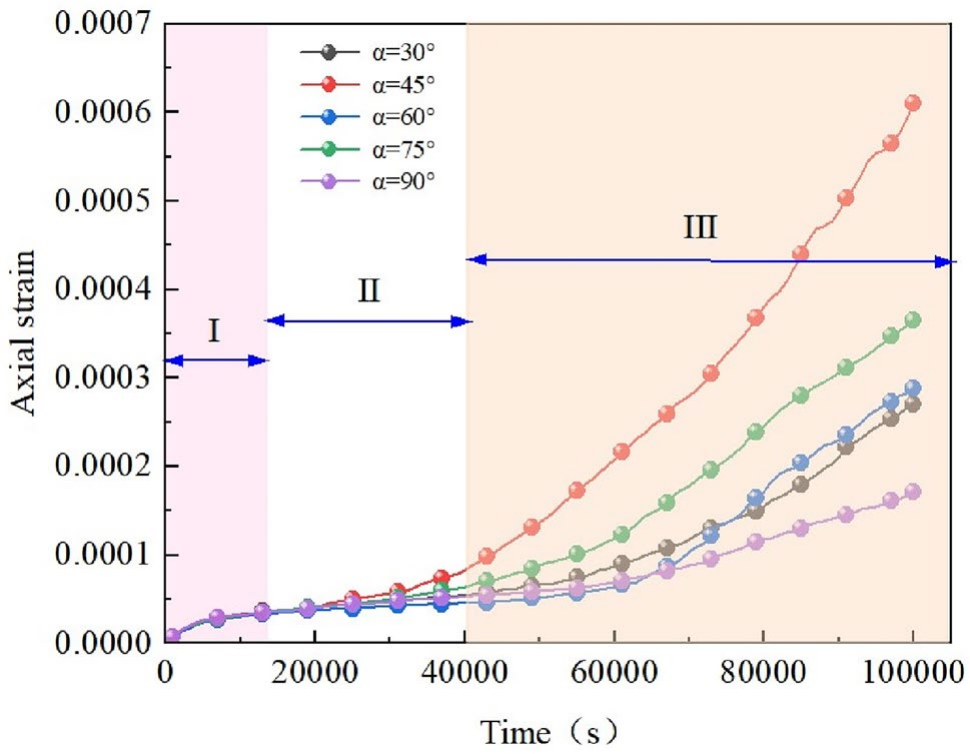
Creep curves of rock mass with different fracture intersection angles.

**Figure 11:**
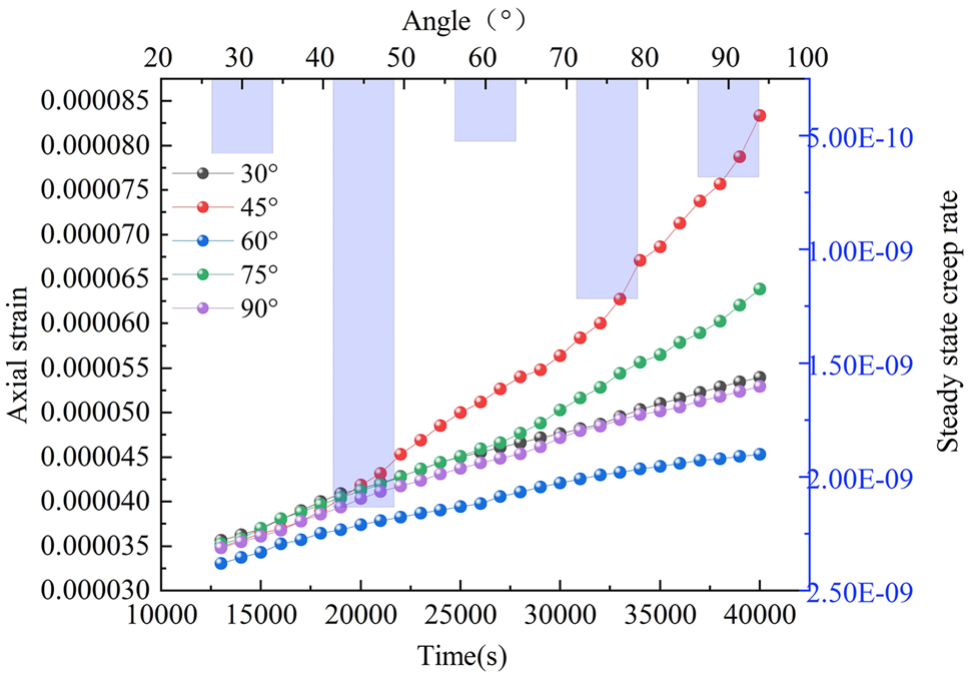
Stages of isotropic creep with different fracture intersection angles.

The length of the X-fractured rock body was taken as 20 mm, and the width was taken as 2 mm. Uniaxial compression creep tests were conducted with various intersection angles between the fracture (30°, 45°, 60°, 75°, 90°) and the material parameters and loading stress held constant. The creep behavior of the rock mass and the equivalent displacement cloud for each intersection angle is shown in Fig. 9. Figure 10 shows the creep curves for different intersection angles. The isotropic creep stages of rock bodies with different fracture intersection angles were analyzed to obtain their corresponding time–displacement curves. The graphs in Fig. 11 shows the isotropic creep stage rates of rock bodies with different fracture intersection angles.

As Fig. 9 shows, the creep equivalent displacement cloud diagrams are quite different from the macroscopic rupture pattern when creep damage occurs in X-fractured rock body with different intersection angles under creep loading. When* α* = 30°, the cracks are mainly tensile–shear mixed cracks, and the wing cracks and shear cracks generated at the left and right ends of the prefabricated main fracture continue to expand and eventually extend along the end of the specimen. The specimen as a whole is in tensile-damage mode. When *α* = 45°, wing cracks are generated at the left and right ends of the main fracture and continue to expand, and compared with *α* = 30°, the cracks at the lower left side of the rock body sprout frequently, and the specimen as a whole is in shear-damage mode. When* α* = 60°, the left and right ends of the main fracture produce anti-flanking cracks and expand to the end of the specimen until complete failure occurs, but as the intersection angle of the fracture increases, the volume of rupture near the prefabricated fracture decreases, and the effect of crack rupture is slightly weakened compared with that at 45°; when* α* = 75°, the main fracture produces wing cracks and eventually shows final H-type damage. When *α* = 90°, the volume of cracking near the prefabricated fracture increases. As the fracture intersection angle increases, the rupture volume near the fracture first decreases, then increases, and finally shows final H-type shear damage.

As Figs. 10 and 11 show, the trends of creep curves corresponding to different slit intersection angles are roughly the same. Both go through the decelerating creep stage (I), enter the isochronous creep stage (II), and finally arrive at the accelerating creep stage (III). The slit intersection angle has little effect on the decelerating creep stage. In the isokinetic creep stage, the steady-state creep rate varies considerably with the creep angle. At 30°, the creep rate is 8.133 × 10^−10^/s; at 45°, the creep rate is 2.133 × 10^−9^ s; at 60°, the creep rate is 5.233 × 10^−10^/s; at 75°, the creep rate is 1.218 × 10^−9^/s; at 90°, the creep rate is 6.814 × 10^−10^/s. In the range of fracture intersection angle from 30° to 90°, the creep rates at 45° and 90° are larger, and the gap is larger.

### Effects of sub-fracture length on creep rupture

**Figure 12:**
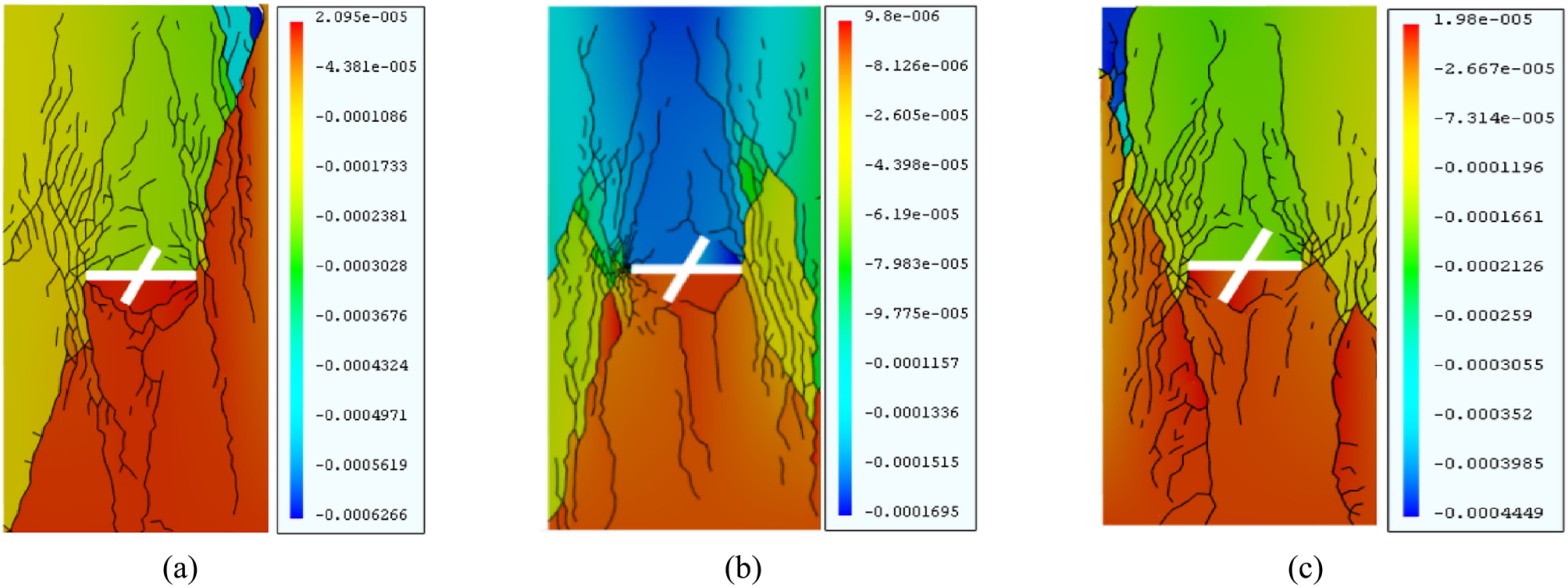
Equivalent displacement maps of the rock mass for different sub-fracture lengths under GDEM. (**a**) h = 12. **b**) h = 14. (**c**)h = 16.

As Fig. 12 shows, as the length of the sub-fracture increases, the cracks generated at the end of the prefabricated cracks have roughly the same form, and the final rupture mode is consistent with mixed tensile–shear damage. When *h* = 12 mm, both the left and right ends of the main fracture produce wing cracks, accompanied by a small number of shear cracks. Secondary cracks are produced by the end of the sub-fracture and form a penetration with the cracks produced around the main fracture. When *h* = 14 mm, wing cracks are produced at the left end of the main fracture, anti-flanking cracks appear at the right end, shear cracks are produced at the lower end of the secondary fracture, and the destruction of the lower part of the rock is obvious; when *h* = 16 mm, anti-flanking cracks are produced at the left end of the main fracture, and At *h* = 16 mm, the main fracture produces anti-flanking cracks at the left end and large secondary cracks at the right end, and the obvious damage areas of the rock body appear in the left and lower parts. When the length of the sub-fracture is between 12 and 16 mm, there is little change in the form of crack extension during the creep rupture process.

**Figure 13:**
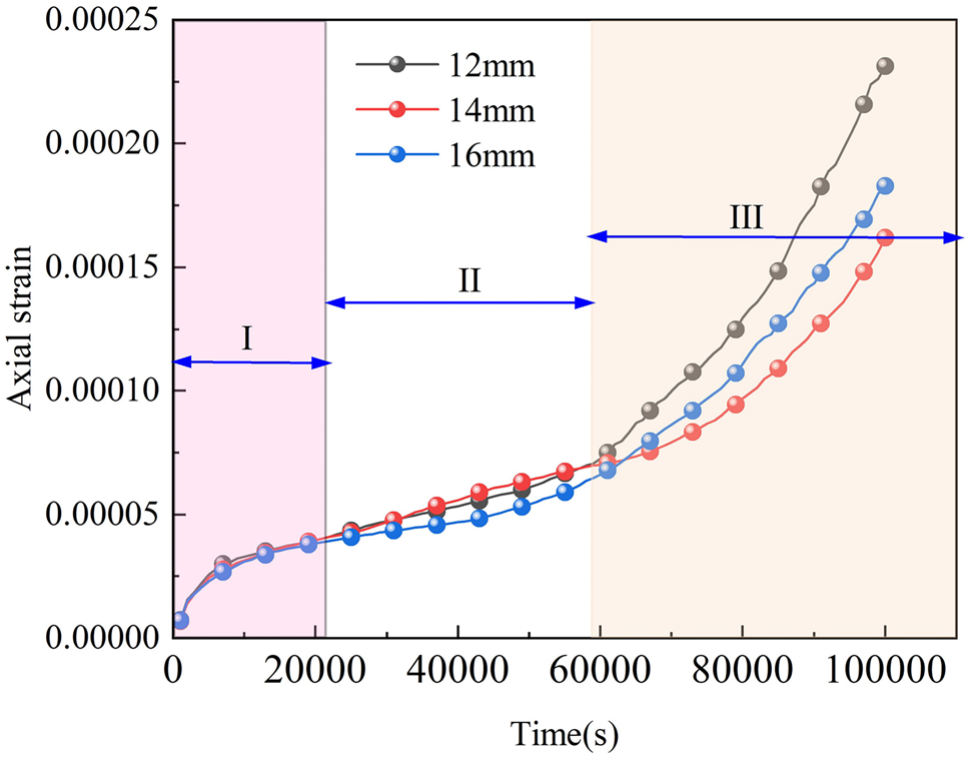
Creep curves of rocks with different sub-fracture lengths.

**Figure 14:**
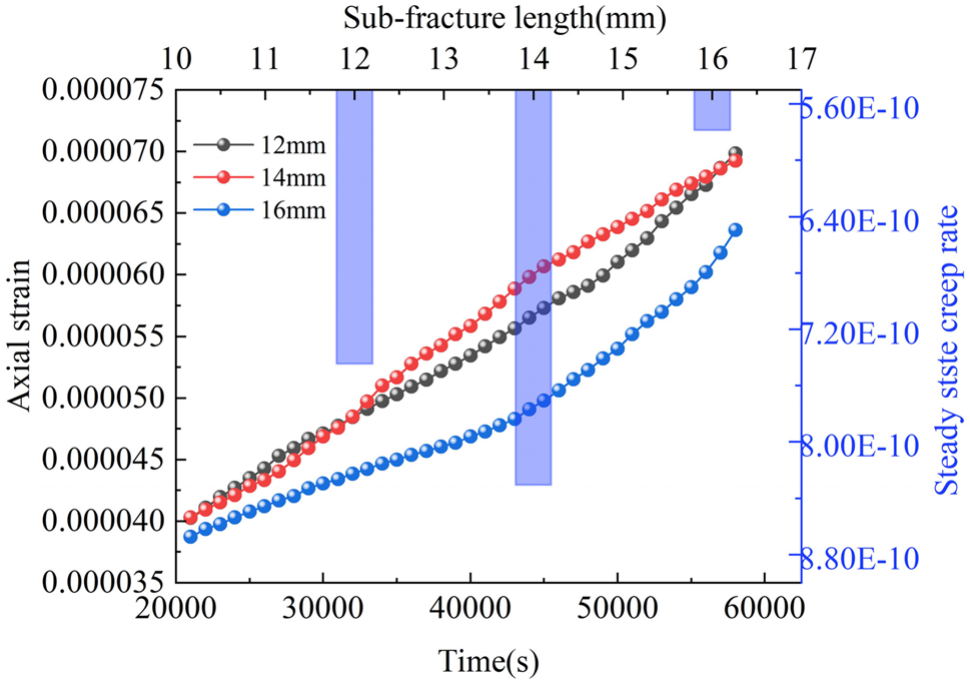
Stages of isokinetic creep for different sub-fracture lengths.

The creep curves (strain curves) of the rock mass at different sub-fracture lengths are shown in Fig. 13. The isotropic creep stages of the rock mass at different sub-fracture angles were analyzed to obtain their corresponding time–displacement curves. The bar graphs in Fig. 14 show the isotropic creep stage rates of the rock mass at each sub-fracture angle.

As shown in Figs. 13 and 14, the curves of the decelerated creep stage (I) of the rock body with different sub-fracture lengths overlap. When the rock body enters the isokinetic creep stage (II), the steady-state creep rate is affected by the sub-fracture length, which shows a trend of increasing and then decreasing, and the rock body with a sub-fracture length of 14 mm is the first one to enter the accelerated creep stage (III). The acceleration curves of the rock body with a sub-fracture length of 14 mm change less compared to the other two. After entering the accelerated creep stage, the accelerated curve of the rock body with a sub-fracture length of 14 mm shows a smaller change in magnitude compared to the other two, and the overall damage degree of the rock body decreases and then increases with the sub-fracture length, in contrast to the changes in the isochronous creep stage. The rock body is more likely to be damaged when the sub-fracture length is 12 mm.

### Effects of variables on creep rupture crack extension

For the rock body with X-fractured, the main fracture was fixed in the horizontal direction. The influence on the creep rupture of the rock body was the greatest when the intersection angle of the fracture was 45° and the smallest when the intersection angle was 90°. The influence was the greatest when the length of the sub-fracture was 12 mm and the smallest when the length of the sub-fracture was 14 mm.

**Figure 15:**
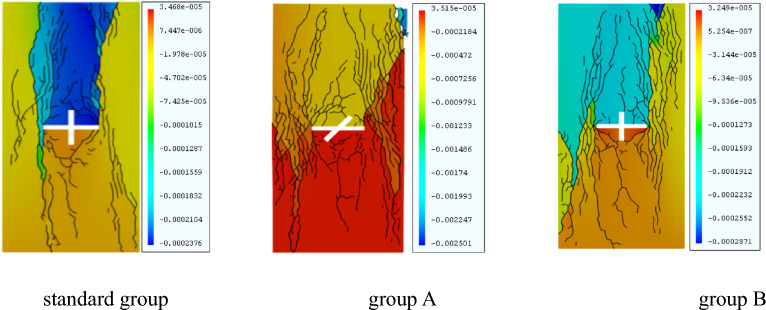
Clouds of rock mass and equivalent displacements under univariate conditions under GDEM.

Therefore, an X-fractured rock body with a 90° intersection angle and 14-mm sub-fracture length was selected as the standard rock body model. Another two groups containing X-fractured rock bodies were considered, and only a single variable was examined for them. Group A had a cleavage intersection angle of 45° and a sub-fracture length of 14 mm, and Group B had a cleavage intersection angle of 90° and a sub-fracture length of 12 mm, as shown in Table 4. The numerical simulation results were compared with the standard rock mass details. The equivalent cloud diagram is shown in Fig. 15.

**Table 4:** Univariate simulation scenarios.

Groups	Main fissure inclination (°)	Intersection angle of fractures (°)	Sub-fracture length (mm)
Standard group	0	90	14
A	0	45	14
B	0	90	12

As Fig. 15 shows, the degree of fracture of the rock body is small in the standard group and group B. In these two groups, the crack volume of group B is relatively large, the crack volume of the standard group is small, the structure is relatively stable, and the degree of fragmentation is small. Group A shows fragmentation of the upper right corner of the rock body during the loading process, and the rock body as a whole has the largest degree of fragmentation and the largest crack volume.

**Figure 16:**
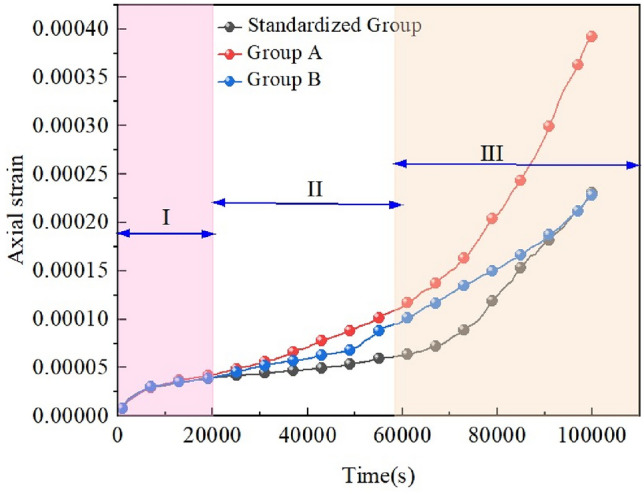
Creep curves for different rock groups.

**Figure 17:**
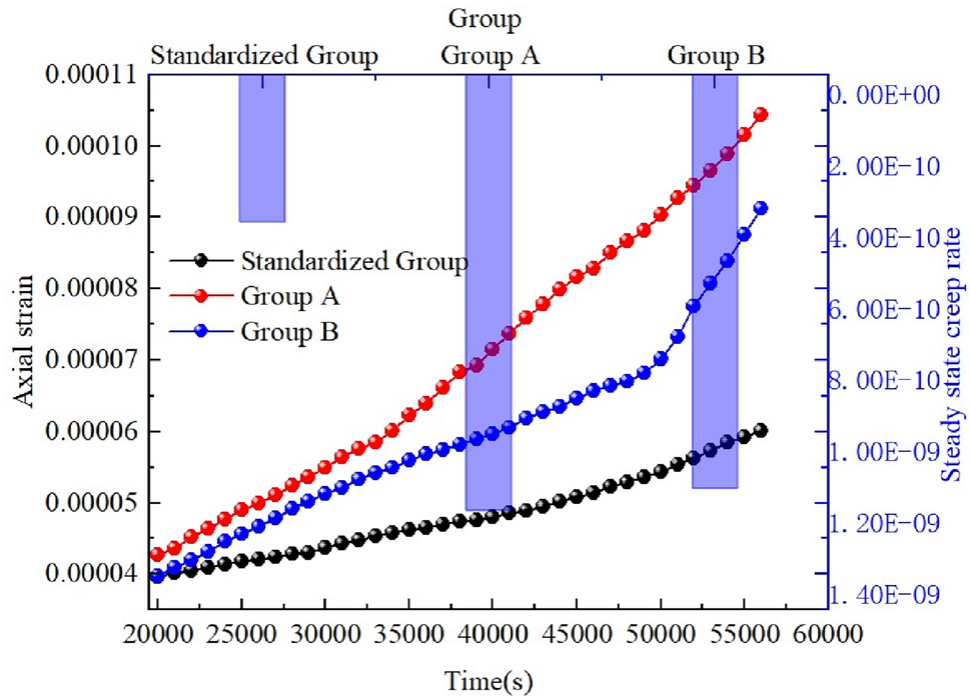
Stages of isokinetic creep in different rock groups.

The creep curves (strain curves) for the different variable groups are shown in Fig. 16 The curves in Fig. 17 show the creep curves for the isotropic creep stages corresponding to the different variable groups, and the bars show the respective creep rates.

The creep curves in Fig. 16 show that a single variable has little effect on the decelerated creep stage (I). As shown in Fig. 17, after entering the isotropic creep stage (II), the steady-state creep rate of the standard group is 4.12 × 10^−10^/s. For group A, i.e., when the fracture intersection angle is changed, the creep rate is 1.22 × 10^−9^/s. For group B, i.e., when the sub-fracture length changes, the creep rate is 1.158 × 10^−9^/s, the fracture intersection angle and sub-fracture length affect the steady-state creep rate, and the difference between the two effects is not significant. In the accelerated creep stage (III), the final strain is consistent over time in group B and the standard group, while the strain is greatly increased in group A compared with the standard group, so the effect of the change in the sub-fracture length on the creep of the rock mass and the effect on the rock mass creep are not significant, and the effect on the rock mass creep is greater when the fracture intersection angle is changed. The change in the intersection angle of the cleavage affects the overall structure of the rock mass, which greatly increases the instability of the structure and thus has a greater impact on the rupture of the rock mass.

## Conclusions

In this paper, the GDEM Continuous–discontinuous software was used to simulate the creep rupture of X-fractured rock body for the purpose of studying the crack extension evolution process and creep rupture of X-fractured rock body under creep load. The following conclusions were obtained:

1. The creep and rupture process of an X-fractured rock body shows that microcosmic crack sprouting, expansion, and penetration manifest macroscopically as creep. In the stage of decelerated creep, the cracks of each wing sprout and expand separately; in the stage of isotropic creep, the cracks of each wing connect and penetrate; in the stage of accelerated creep, a macroscopic rupture surface is formed, and the rock body is damaged.
2. The X-fractured rock body exhibited a mixed tensile–shear damage mode dominated by tensile damage. In the isokinetic creep stage, the steady-state creep rate increased and then decreased with increasing sub-fracture length. The creep rate was greatest when the fracture intersection angle α was 30°.
3. When the length of the sub-fracture was 14 mm, the rock body entered the accelerated creep stage. For different fracture angles, the time order of entering the accelerated creep stage was consistent with the order of the creep rate. When the fracture angle was 45° and the length of the sub-fracture was 12 mm, the rock body was broken to the greatest extent, which has an obvious influence on its creep damage.
4. Changes in the fracture intersection angle and the length of the sub-fracture affect the steady-state creep rate and accelerated creep stage of a rock body containing X-fractured. The influence of the fracture intersection angle on the creep rate is greater than that of the length of the sub-fracture.
5. The results of this study provide valuable reference information for the study of the mechanical properties and creep disaster management of non-parallel fractured rock bodies.

## Data Availability

The datasets used and/or analysed during the current study are available from the corresponding author upon reasonable request.
